# Exclusive Breastfeeding Duration and Perceptions of Infant Sleep: The Mediating Role of Postpartum Anxiety

**DOI:** 10.3390/ijerph19084494

**Published:** 2022-04-08

**Authors:** Siân M. Davies, Bethany F. Todd-Leonida, Victoria M. Fallon, Sergio A. Silverio

**Affiliations:** 1 School of Psychology, Faculty of Health, Liverpool John Moores University, Liverpool L3 3AF, UK; s.m.davies@2020.ljmu.ac.uk; 2 Department of Psychology, Institute of Population Health, Faculty of Health and Life Sciences, University of Liverpool, Liverpool L69 7ZA, UK; b.f.todd-leonida@liverpool.ac.uk (B.F.T.-L.); v.fallon@liverpool.ac.uk (V.M.F.); 3 Department of Women & Children’s Health, School of Life Course & Population Sciences, Faculty of Life Sciences & Medicine, King’s College London, London SE1 7EH, UK

**Keywords:** exclusive breastfeeding, postpartum anxiety, perceptions of infant sleep, mediation analysis

## Abstract

(1) Background: Existing literature has identified associations between exclusive breastfeeding, maternal mental health, and infant sleep. This study aims to examine these relationships simultaneously and consider the mediating role of postpartum anxiety. (2) Methods: Participants completed validated measures of postpartum anxiety, infant sleep, and reported exclusive breastfeeding duration. Postpartum mothers with infants between six and twelve months (*n* = 470) were recruited to a cross-sectional online survey containing a battery of psychological measures. (3) Results: Correlation analyses examined the relationships between the predictor (exclusive breastfeeding duration), outcome (perceptions of infant sleep), and mediator (postpartum anxiety). Exclusive breastfeeding duration was significantly associated with postpartum anxiety (*p* < 0.05), postpartum anxiety was significantly associated with perceptions of infant sleep (*p* < 0.001), and exclusive breastfeeding duration was significantly associated with perceptions of infant sleep (*p* < 0.001). A simple mediation model was conducted, showing a significant total (B = −0.029 (0.010), *p* < 0.05), direct (B = −0.035 (0.009), *p* < 0.001), and indirect effect (B = 0.007, SE = 0.003, 95% CI = 0.000 to 0.014) of exclusive breastfeeding duration on perceptions of infant sleep via postpartum anxiety. (4) Conclusions: Associations were identified between exclusive breastfeeding duration, postpartum anxiety, and perceptions of infant sleep. The mediation model suggests postpartum anxiety may be an underlying mechanism which reduces exclusive breastfeeding duration and negatively affects maternal perceptions of infant sleep quality.

## 1. Introduction

### 1.1. Exclusive Breastfeeding Duration and Postpartum Anxiety

Research has demonstrated that common mental disorders have steadily increased in women over the years, with onset of depression and anxiety being more likely to occur at childbearing age than any other time of life [1]. Postnatal depression (PND) has received more research attention in the field of perinatal mental health; however, more recent evidence suggests postpartum anxiety (PPA) exists as a distinct condition and may occur at a higher rate than PND [2,3]. Existing studies have demonstrated associations between PPA and various negative maternal and infant health outcomes, including impaired maternal-infant bonding [4], perceptions of difficult infant temperament [5], reduced maternal self-efficacy [6], and poor infant feeding outcomes [7].

The World Health Organization (WHO) recommends mothers should exclusively breastfeed their infants for the first six months of life to promote optimal health and development [8]. The UK National Health Service (NHS) echoes this advice, emphasizing the numerous health benefits for both mother and infant [9]. Despite the evidence-base supporting the benefits of exclusive breastfeeding, approximately 81% of women in the UK initiate, however these rates drop to 34% by six months, and 0.5% by twelve months [10]. Furthermore, 90% of women who discontinue breastfeeding do so before they are ready [11]. This intention-behaviour gap has been consistently associated with sub-optimal mental health outcomes such as guilt and shame [12], depression [13], and anxiety [14].

Existing research suggests maternal anxiety influences infant feeding outcomes, with both PND and PPA having a significant negative effect on relationships with breastfeeding initiation, duration, and exclusivity [14,15]. Early breastfeeding discontinuation, or failure to meet breastfeeding intentions, has also been associated with feelings of anxiety [16,17] and stigma and guilt [12,18] with regards to the health and development of the infant. Thus, it remains unclear whether postpartum anxiety is an antecedent or consequence of sub-optimal infant feeding outcomes. It is likely that the relationship is transactional in nature and therefore it is plausible that a mother’s experiences with infant feeding may influence her current feelings of anxiety or vice versa.

### 1.2. Postpartum Anxiety and Perceptions of Infant Sleep

Infant sleep cycles are polyphasic throughout both day and night during early development, in order for infants to wake at regular intervals to feed [19]. During early infancy, frequent night wakings are considered to be a typical, adaptive occurrence [20]. Sleep-wake regulation is considered to be a significant developmental task during the first year of life, and an infant’s circadian rhythm evolves and becomes established with maturity [21]. However, despite recurrent wakings being a normative aspect of infant development, this period can still place great demand on mothers [22]. Sleep, or lack thereof, is one of the most challenging aspects of the postpartum period to navigate, with mothers frequently citing insufficient, inefficient, or fragmented sleep, and poor perceptions of infant sleep quality [23] affecting maternal mood [24,25,26], which can lead to maternal fatigue, a recognised precursor to anxious and depressive symptomology during the postpartum period [27,28].

This relationship has been conceptualised as bi-directional, with biological explanations for the relationship between negative maternal mental health and infant sleep [29]. Mothers who have experienced persistent anxiety throughout pregnancy may produce higher levels of stress hormones such as cortisol and adrenaline. This can impact the intrauterine environment and expose the infant to unhealthy levels of these hormones and thus impact brain development. Existing literature has found that chronic and extreme stress and anxiety during pregnancy are linked to early infant regulatory issues such as irregular sleep patterns [30,31]. Another theoretically viable explanation for this relationship lies in maternal perceptions of infant sleep which may be obscured by poor maternal mental health. For instance, mothers with high levels of anxiety may be vulnerable to misinterpreting their infants’ behaviours [32], and therefore perceive typical infant waking as atypical [33,34]. It is therefore plausible that infant sleep duration is perceived more negatively due to impaired maternal mood.

### 1.3. Exclusive Breastfeeding and Perceptions of Infant Sleep

There is a common lay belief that breastfeeding is associated with poor sleep due to breast milk digesting at a quicker rate than formula [35]. This may result in pre-conceived notions regarding the relationship between feeding method and infant sleep quality, with mothers anticipating that should they breastfeed, their infant is more likely to have fragmented or insufficient sleep [36]. However, there appears to be conflicting narratives surrounding breastfeeding, with other evidence suggesting that breastfeeding mothers may perceive this fragmented sleep as typical and therefore may be more accepting of night-wakings and have a more positive perception of their infant’s sleep [37].

Previous research exploring the mediating role of PPA in relation to maternal-infant bonding, PPA, and perceptions of infant temperament, provides a key rationale for investigating the role maternal anxiety can play in other domains of maternal and infant well-being and behaviour [5]. The relationship between exclusive breastfeeding (EBF) duration and perceptions of infant sleep appears to be bi-directional and dynamic in nature, with existing literature demonstrating clear associations between all three constructs (EBF duration, postpartum anxiety, and perceptions of infant sleep [38]). Another important rationale for this study is that PPA is modifiable, and therefore if it does mediate the relationship between EBF duration and perceptions of infant sleep, there may be scope for interventions designed to target maternal anxiety which can then consequently improve EBF experiences and also maternal perceptions of their infants’ sleep.

### 1.4. Aims

The aim of this study is therefore to examine PPA, EBF duration, and perceptions of infant sleep simultaneously. A secondary aim of the present study is to examine whether PPA mediates the relationship between EBF duration and perceptions of infant sleep. The present study has the following hypotheses:

1. There will be a negative association between PPA and EBF duration
2. There will be a negative association between PPA and perceptions of infant sleep
3. There will be an association between EBF duration and perceptions of infant sleep
4. PPA will mediate the relationship between EBF duration and perceptions of infant sleep

## 2. Materials and Methods

### 2.1. Participants

Eligibility for participation in the survey was based on the following inclusion criteria: mothers aged ≥ 18 years with infants aged between six and twelve months, who had been born at term gestation or thereafter (defined as 37 + weeks). These narrow criteria were due to evidence suggesting preterm infants may experience delays in developmental functioning [39]. Recruitment was conducted through online social media platforms and participants were provided a link to the online survey hosted by Qualtrics. Participants (*n* = 470) were predominately white (92%), married (67%), homeowners (46%), and educated to a university level (46%), with a professional occupation (30%). Some mothers reported a current clinical diagnosis of anxiety (18%) and/or depression (13%), which is comparable to UK prevalence rates. See Table 1 for full demographic characteristics.

### 2.2. Ethics

Ethical approvals were sought from and granted by the University of Liverpool Research Ethics Committee (ref: IPHS/2022).

### 2.3. Design and Procedure

A cross-sectional online survey using a battery of questionnaires was administered to participants who were recruited through social media platforms (Facebook, Twitter) via advertisements that provided a link to the Qualtrics survey platform. This included a demographic questionnaire, with a EBF duration item, and the following measures: The Brief Infant Sleep Questionnaire (BISQ) [40] and the Postpartum Specific Anxiety Scale (PSAS) [41]. Prior to commencing the study, participants were instructed to read an information sheet and electronically sign the consent form. Eligible participants were permitted to enter the main questionnaire, whilst those deemed ineligible were then screened to an exit message. A full electronic debrief was provided on completion of the survey and participants were then redirected to an optional draw to receive a £25 shopping voucher should they wish.

### 2.4. Measures

#### 2.4.1. Demographics

Maternal and infant characteristics included maternal age, country of residence, marital status, occupational status, educational attainment, household size, home-owner status, and current clinical diagnosis of anxiety and/or depression. Birth order, gestational age at birth, multiple birth status, and feeding practices were also recorded (see Table 1).

#### 2.4.2. Exclusive Breastfeeding Duration

To assess EBF duration in the first six months of life, mothers were asked, ‘For how long have you exclusively breastfed this infant? (This includes expressed breast milk in a bottle). If you no longer exclusively breastfeed, for how many weeks did you exclusively breastfeed for?’. Any mothers who exceeded 26 weeks of breastfeeding were recoded as 26 weeks.

#### 2.4.3. The Brief Infant Sleep Questionnaire (BISQ)

The BISQ [40] is a 29-item measure scored on a Likert scale, used to determine infant sleep behaviours and patterns during the previous seven days. For the purposes of this study, a sleep quality variable was generated by computing three of the items: ‘Difficulty in bedtime routine’ (1 = very easy, 5 = very difficult); ‘Perceptions of sleep quality’ (1 = very well and 6 = very poorly); and ‘Perceptions of sleep problem’ (1 = a very serious problem and 3 = not a problem at all). Items 1 and 2 were reverse scored, as higher scores indicate more positive perceptions of infant sleep quality. The rationale for the items selected was to create a subjective measure of sleep quality which was appropriate for a sample of infants aged 6–12 months. Some objective items in the BISQ, such as ‘total sleep duration’ were not considered appropriate due to the broad age range of infants in the sample. In addition, items such as ‘exact bedtime routine’ may not be indicative of sleep difficulties, as some mothers may not perceive flexible bedtimes as problematic. The composite sleep quality variable demonstrated average reliability—McDonald’s ω = 0.79.

#### 2.4.4. Postpartum-Specific Anxiety Scale (PSAS)

The PSAS [41] is used to establish the incidence of maternal- and infant-focused anxieties experienced during the previous seven days. It contains 51 items within four different constructs. ‘Maternal competence and attachment anxieties’ (15 items) relate to anxieties regarding maternal self-efficacy, parental competence, and the maternal-infant relationship. ‘Infant safety and welfare anxieties’ (11 items) relate to fears of infant illnesses, accidents, and cot death. ‘Practical infant care anxieties’ (7 items) relate to anxieties surrounding infant care such as feeding, sleep, and general routine. ‘Psychosocial adjustment to motherhood’ (18 items) relates to postpartum adjustment anxieties regarding the management of personal appearance, relationships, support, work, finances, and sleep. Answers are given a score ranging from 1 to 4 with the maximum score being a total of 204. Scores of 112 or above are suggestive of clinical levels of anxiety. The PSAS demonstrated excellent reliability, McDonald’s ω = 0.95.

### 2.5. Method of Analysis

Correlation analyses were initially conducted to investigate the associations between EBF duration, PPA, and perceptions of infant sleep. Following this, a simple mediation analysis to examine the mediating role of PPA was performed using PROCESS macro for SPSS 24 (IBM, New York, NY, USA). The significance of the indirect effects was tested using bootstrapping procedures. Unstandardized indirect effects were computed for each of 5000 bootstrapped samples, and the 95% confidence intervals were reported.

## 3. Results

Correlation analyses were initially conducted to investigate the associations between EBF duration, PPA, and perceptions of infant sleep (see Table 2).

### Simple Mediation Analysis Examining the Relationship between Exclusive Breastfeeding Duration and Perceptions of Infant Sleep via Postpartum Anxiety

**Figure 1:**
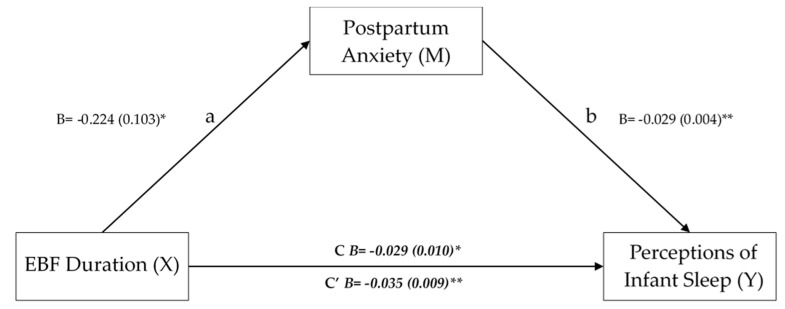
A simple mediation showing the total (C), direct (C’), and indirect effects of exclusive breastfeeding (EBF) duration on perceptions of infant sleep via postpartum anxiety after controlling for maternal age, socioeconomic status, birth order, and marital status. * = *p* < 0.05; ** *p* < 0.001.

A simple mediation analysis was conducted to test whether the relationship between EBF duration and perceptions of infant sleep was mediated by PPA after controlling for maternal age, SES, birth order, and marital status. As Figure 1 demonstrates, the association between EBF duration and PPA was statistically significant, as was the association between PPA and perceptions of infant sleep. Furthermore, the model found a significant indirect effect of EBF duration on perceptions of infant sleep via PPA (B = 0.007, SE = 0.003, 95% CI = 0.000 to 0.014).

## 4. Discussion

### 4.1. Summary of Main Findings

The objective of the current study was to examine the relationships between EBF duration, PPA, and perceptions of infant sleep. Furthermore, we were interested to understand the mediating role of PPA and how it may influence the relationship between EBF duration and perceptions of infant sleep. Results supported all predicted associations between EBF duration and PPA (Hypothesis 1); postpartum anxiety and perceptions of infant sleep (Hypothesis 2); and EBF duration and perceptions of infant sleep (Hypothesis 3). The mediation analysis found a significant total effect, direct effect, and indirect effect of EBF duration on perceptions of infant sleep via PPA (Hypothesis 4).

The results from the individual regressions are consistent with previous literature. Firstly, EBF duration was negatively associated with PPA, with mothers who exclusively breastfed for shorter durations in the first six months displaying higher levels of PPA at 6–12 months. This is concurrent with a systematic review of 33 studies, which demonstrated that increased levels of anxiety are associated with shorter EBF duration [14]. This relationship may begin during pregnancy with another systematic review finding high levels of prenatal anxiety were associated with breastfeeding exclusivity [42]. A number of studies have found that anxiety is positively associated with breastfeeding self-efficacy [14,43]. Routinely assessing anxiety symptoms after birth and providing additional breastfeeding support to mothers experiencing higher levels of anxiety may be beneficial in supporting both mental health and the continuation of breastfeeding.

Secondly, PPA was negatively associated with perceptions of infant sleep with poorer perceptions of infant sleep being correlated with higher levels of PPA. This supports existing literature which demonstrates that maternal mental health can alter perceptions of infant sleep quality [32]. Research emphasises that exchanges between mother and infant are reciprocal in nature and so therefore adverse emotional disturbances can then result in an escalation of negative maternal-infant interactions [44]. This may provide an explanation as to why mothers with PPA may perceive their infants’ sleep as problematic, as negative affective states may skew how a mother interprets her infant’s behaviour and development, which in turn may exacerbate the mother’s mental health [32]. Previous work has also highlighted associations between maternal emotional availability at bedtime and ratings of infant sleep difficulties [45,46]. Therefore, excessive anxiety may reduce a mother’s emotional availability during bedtime and therefore distort subsequent perceptions of infant sleep quality and quantity.

Finally, results identified a negative association between EBF duration and perceptions of infant sleep quality. Specifically, women with shorter exclusive breastfeeding duration had more positive perceptions of infant sleep at six to twelve months. However, a body of work in the last decade finds an inconclusive relationship between feeding method and infant sleep, with some studies using objective measurement, finding that breastfed infants sleep as much or more than formula fed infants [37]. Contrastingly, other work examining subjective perceptions of sleep demonstrates that mothers who supplement with formula perceive better sleep quality [35]. Therefore, although objective quantity of sleep may not vary by feeding method, subjective perceptions of sleep quality may be a more pertinent predictor of EBF duration.

To our knowledge, a mediation analysis exploring these variables simultaneously has never been conducted, with results providing a unique insight into how anxiety influences the relationship between EBF duration and perceptions of infant sleep. Our findings indicate that PPA strengthens the association between reduced EBF duration and positive perceptions of infant sleep quality, indicating a suppression effect. This occurs when the mediator increases the predictive validity of another variable (i.e., EBF duration). Therefore, when higher levels of anxiety are experienced in mothers, the relationship between earlier breastfeeding cessation and positive perceptions of sleep quality is magnified. This indicates that symptoms of anxiety are important to consider in any study which examines feeding method and infant sleep. It also suggests that if PPA symptoms are quickly identified and reduced by appropriate support interventions, this may then improve breastfeeding outcomes and consequently the manner in which a mother perceives her infant’s sleep quality.

### 4.2. Strengths, Limitations, and Future Directions

This study does not infer causation; however, it does offer interesting findings in that PPA may be an underlying mechanism to explain the relationship between shorter duration of EBF and positive perceptions of infant sleep. Future work should consider looking at this mechanism longitudinally. Whilst we endeavoured to create an age-appropriate sleep variable by only selecting items from the BISQ that would be universally problematic to mothers with infants 6–12 months, the reliability of this measure was average. There is an absence of valid and reliable sleep measures in the literature, and future work should consider the development and validation of a new subjective measure of infant sleep. Qualitative work to ascertain what items are acceptable and pertinent to mothers should precede this. As commonly found in studies of this nature, there are usually a significantly high proportion of exclusively breastfeeding mothers that participate in research compared to exclusively formula feeding mothers [14]. This is likely to influence findings and therefore replication of findings in larger samples that include higher quantities of mothers using alternative feeding methods would be beneficial. A key strength of the present study compared to previous work exploring the relationship between PPA and perceptions of infant sleep is the use of childbearing-specific measures of maternal mood. There is a growing body of evidence that highlights their efficacy in accurately measuring perinatal anxiety and predicting maternal and infant health outcomes, such as infant feeding [7] and maternal bonding [4].

Existing studies have demonstrated associations between perceived maternal competence and infant feeding difficulties with reduced feelings of competency resulting in early breastfeeding cessation [47]. Furthermore, recent evidence suggests that parenting literature that focuses heavily on strict routine can often result in the attenuation of parenting confidence [48]. Therefore, it is possible that mothers who implement sleep and feeding routines may not have realistic expectations of infant developmental milestones such as cluster feeding and sleep regressions. This may result in reduced feelings of self-efficacy and consequently negative perceptions of breastfeeding behaviour and sleep quality. Further investigation should consider the relationship between maternal self-efficacy and anxieties surrounding infant routine care. Enhancing confidence and normalising infant development may reduce idealistic expectations and perceptions of infant behaviour.

## 5. Conclusions

The present study found associations between EBF duration and PPA, and PPA and perceptions of infant sleep. Furthermore, the mediation analysis identified a significant total, direct, indirect, and suppression effect between infant feeding and perceptions of infant sleep via PPA. Identification and management of maternal and infant focused anxieties may improve breastfeeding outcomes and perceptions of infant sleep quality.

## Figures and Tables

**Table 1:** Maternal and Infant Demographics (*n* = 470).

Maternal Characteristic	Value	Infant Characteristic	Value
Maternal age (mean years ± SD)	32.22 (4.86)	Birth Order (*n*/%)	
Ethnicity (*n*/%)		Oldest	39 (8.3)
White	439 (93.4)	Middle	1 (0.2)
Black African	1 (0.2)	Youngest	188 (40.0)
Chinese	2 (0.4)	Only	241 (51.3)
Indian	2 (0.4)	Timing of Birth (*n*/%)	
Black Other	2 (0.4)	Full Term (>37 <40 weeks)	358 (76.0)
Other	21 (4.5)	Late Term (>41 <42 weeks)	112 (23.8)
Prefer not to say	3 (0.6)	Multiple Birth	1 (0.2)
Marital Status (*n*/%)		Breastfeeding Initation (*n*/%)	561 (99.8)
Married	315 (67.0)	Yes	388 (82.6)
Co-habiting	133 (28.3)	No	82 (17.4)
Single	22 (4.7)		
Occupation (*n*/%)		**Maternal and Infant Behaviours (mean ± SD)**	
Managers, Directors and Senior Officials	42 (8.9)	Exclusive Breastfeeding Duration (weeks)	17.67 (10.68)
Professionals	211 (44.9)	Postpartum Anxiety	104.29 (23.85)
Associate Professionals and Technical	9 (1.9)	Infant Sleep	10.86 (2.26)
Administrative and Secretarial	44 (9.4)	Postpartum Anxiety	104.29 (23.85)
Skilled Trade	7 (1.5)		
Caring, Leisure and Other Service	42 (8.9)		
Sales and Customer Service	43 (9.1)		
Process, Plant and Machine Operatives	1 (0.2)		
Elementary	10 (2.1)		
Not in Paid Occupation	61 (13.0)		
Education Attainment (*n*/%)			
Postgraduate education	139 (29.6)		
Undergraduate education	214 (45.5)		
A-Levels or college equivalent	66 (14.0)		
GCSEs or secondary school equivalent	26 (5.5)		
No qualifications	6 (1.3)		
Other qualification	19 (4.0)		
Living Status (*n*/%)			
Own property	325 (69.1)		
Rent privately	95 (20.2)		
Rent from local authority	26 (5.5)		
Live with parents	14 (3.0)		
Other	10 (2.1)		
Household Size (inc. participant) (*n*/%)			
2 people	23 (4.9)		
3 people	249 (53.0)		
4 people	143 (30.4)		
5 people	36 (7.7)		
6 or more people	19 (4.0)		
Current Diagnosis of Anxiety (*n*/%)			
Yes	83 (17.7)		
No	386 (82.1)		
Prefer not to say	1 (0.2)		
Current Diagnosis of Depression (*n*/%)			
Yes	63 (13.4)		
No	406 (86.4)		
Prefer not to say	1 (0.2)		

**Table 2:** Descriptive statistics and Pearson’s correlations for exclusive breastfeeding duration, postpartum anxiety, and perceptions of infant sleep.

Variable	Mean (±SD)	Postpartum Anxiety	Perceptions of Infant Sleep
Exclusive Breastfeeding Duration	17.67 (10.68)	−0.13 *	−0.16 **
Postpartum Anxiety	104.29 (23.85)	–	−0.26 **
Perceptions of Infant Sleep	10.86 (2.26)	–	–

* = *p* < 0.05; ** = *p* < 0.001.

## Data Availability

The data that support the findings of this study are available on request from the corresponding author. The data are not publicly available due to privacy or ethical restrictions.

## Abbreviations

BISQ—Brief Infant Sleep Questionnaire; EBF—Exclusive Breastfeeding; NHS—National Health Service; PPA—Postpartum Anxiety; PND—Postnatal Depression; PSAS—Postpartum Specific Anxiety Scale; WHO—World Health Organization.
